# Multi-Biomarkers for Early Detection of Type 2 Diabetes, Including 10- and 12-*(Z*,*E)*-Hydroxyoctadecadienoic Acids, Insulin, Leptin, and Adiponectin

**DOI:** 10.1371/journal.pone.0130971

**Published:** 2015-07-01

**Authors:** Aya Umeno, Kohzoh Yoshino, Yoshiko Hashimoto, Mototada Shichiri, Masatoshi Kataoka, Yasukazu Yoshida

**Affiliations:** 1 Health Research Institute (HRI), National Institute of Advanced Industrial Science and Technology (AIST), 2217–14 Hayashi-cho, Takamatsu, Kagawa 761–0395, Japan; 2 Health Research Institute (HRI), National Institute of Advanced Industrial Science and Technology (AIST), 1-8-31 Midorigaoka, Ikeda, Osaka 563–8577, Japan; USDA-ARS, UNITED STATES

## Abstract

We have previously found that fasting plasma levels of totally assessed 10- and 12-*(Z*,*E)*-hydroxyoctadecadienoic acid (HODE) correlated well with levels of glycated hemoglobin (HbA1c) and glucose during oral glucose tolerance tests (OGTT); these levels were determined via liquid chromatography—mass spectrometry after reduction and saponification. However, 10- and 12-*(Z*,*E)*-HODE alone cannot perfectly detect early impaired glucose tolerance (IGT) and/or insulin resistance, which ultimately lead to diabetes. In this study, we randomly recruited healthy volunteers (n = 57) who had no known history of any diseases, and who were evaluated using the OGTT, the HODE biomarkers, and several additional proposed biomarkers, including retinol binding protein 4 (RBP4), adiponectin, leptin, insulin, glycoalbumin, and high sensitivity-C-reactive protein. The OGTT revealed that our volunteers included normal individuals (n = 44; Group N), “high-normal” individuals (fasting plasma glucose 100–109 mg/dL) with IGT (n = 11; Group HN+IGT), and diabetic individuals (n = 2; Group D). We then used these groups to evaluate the potential biomarkers for the early detection of type 2 diabetes. Plasma levels of RBP4 and glycoalbumin were higher in Group HN+IGT, compared to those in Group N, and fasting levels of 10- and 12-*(Z*,*E)*-HODE/linoleic acids were significantly correlated with levels of RBP4 (*p* = 0.003, r = 0.380) and glycoalbumin (*p* = 0.006, r = 0.316). Furthermore, we developed a stepwise multiple linear regression models to predict the individuals’ insulin resistance index (the Matsuda Index 3). Fasting plasma levels of 10- and 12-*(Z*,*E)*-HODE/linoleic acids, glucose, insulin, and leptin/adiponectin were selected as the explanatory variables for the models. The risks of type 2 diabetes, early IGT, and insulin resistance were perfectly predicted by comparing fasting glucose levels to the estimated Matsuda Index 3 (fasting levels of 10- and 12-*(Z*,*E)*-HODE/linoleic acids, insulin, and leptin/adiponectin).

## Introduction

Early detection and treatment of diabetes can postpone, or even prevent, the serious complications that are associated with diabetes (e.g., blindness, amputation, and renal disease). The criteria for diagnosing diabetes is a fasting plasma glucose (FPG) concentration of >126 mg/dL and a plasma concentration of >200 mg/dL at 120 min after the oral glucose tolerance test (OGTT). In comparison, healthy individuals have fasting or 120 min post-OGTT FPG concentrations of <110 and <140 mg/dL, respectively. The Japan Diabetic Society recommends that subjects with an FPG value of 100–109 mg/dL be classified as “high-normal” in the range of glucose metabolism disorders, and that subjects with a “high-normal” FPG value should undergo a 75-g OGTT to determine whether they are normal, borderline, or diabetic [1]. Therefore, diabetes prevention requires the identification of persons who are borderline (both impaired glucose tolerance [IGT] and impaired fasting glycemia [IFG]) or “high-normal” (do not have diabetes or are not healthy).

Insulin resistance and homeostasis are also important topics when discussing the risk of diabetes. The homeostasis model assessment of insulin resistance (HOMA-IR) and Matsuda Index have both been developed to quantify insulin resistance. HOMA-IR is calculated as (FPG × fasting insulin)/405 (normal level, <1.6; insulin resistance, >2.5; according to the Japan Diabetes Society). The Matsuda Index is calculated using plasma glucose and insulin concentrations during the OGTT (Matsuda index 3 = 10,000/square root of [fasting glucose × fasting insulin] × [mean glucose × mean insulin during the OGTT] (0, 60, 120 min, respectively; normal level, >3) [2]. Therefore, both glucose tolerance and insulin homeostasis are important factors in evaluating diabetic risk and in maintaining human health. However, as the OGTT is a time-consuming and optional test in Japan, few people who undergo the OGTT each year. Furthermore, when the OGTT is performed, the glucose levels at 120 min after the OGTT are occasionally measured without insulin data, which results in the lack of information regarding insulin homeostasis.

Fat accumulation in the human body releases several adipokines from adipocytes, and it is known that some of these adipokines elevate insulin resistance, cause metabolic syndrome, and promote type 2 diabetes. Several biomarkers have been examined and used for the prediction of diabetes. For example, adiponectin [3], leptin [3,4], glycoalbumin [5], and retinol binding protein 4 (RBP4) [6,7] have all been proposed, along with the well-known markers: glycated hemoglobin (HbA1c) and insulin. Adiponectin and leptin are relatively prevalent, and are occasionally used in diabetes screening. Glycated albumin (glycoalbumin) is expressed as the percentage of serum glycated albumin in the total serum albumin, and reflects shorter-term glycemic control (compared to HbA1c), as albumin is reduced by 50% within 2–3 weeks [5]. RBP4 is another adipocyte-derived factor, which is primarily produced in the liver (approximately 20% of all circulating RBP4) and acts on muscle and/or liver via mechanisms that are either retinol-dependent or independent. It has also been recently reported that RBP4 is involved in the early phases of developing adiposity and insulin resistance [8].

Oxidative stress is a common pathogenic factor that is thought to lead to insulin resistance, β-cell dysfunction, IGT, and IFG. Products of lipid peroxidation have received considerable attention, as they serve as indices for oxidative stress, given that lipids are susceptible to oxidation *in vivo*. As a result, various lipid products have been evaluated, using diverse methods and techniques. For example, F_2_-isoprostanes consist of a series of chemically stable prostaglandin F_2_-like compounds that are formed independent of the cyclooxygenase pathway, and have been assessed as the gold standard for evaluating oxidative stress *in vivo* [9,10]. In contrast, hydroxyoctadecadienoic acids (HODEs) [11–14] are derived from linoleic acids (LA), and have also attracted attention, with some reports describing the detection of these molecules *in vivo*. For example, some studies have evaluated the formation of 9-hydroxyoctadecadienoic acid in the erythrocyte membranes of patients with diabetes [12], and hydroxyl-fatty acids that are derived from LA in low-density lipoproteins from patients with atherosclerosis [11]. Furthermore, the use of HODEs as biomarkers has been reported in the recent literature [15–20]. Moreover, we have also recently reported that fasting levels of 10- and 12-*(Z*,*E)*-HODE, although not 9- and 13-*(E*,*E)*-HODE (these are free radical-mediated specific products), exhibited significant correlation with plasma levels of HbA1c, glucose, insulin secretion, and resistance index [21]. However, 10- and 12-*(Z*,*E)*-HODE alone cannot perfectly predict the risk of diabetes.

Therefore, in this study, we advanced our previous work and focused on the detection of early-stage IGT and “high-normal” states, as well as insulin homeostasis abnormality during fasting, without using the OGTT. Instead, we used several fasting biomarkers, especially 10- and 12-(*Z*,*E*)-HODE, for the early detection of these conditions. Thus, we randomly collected data from healthy volunteers who had not received any specific diagnoses of diabetes or other illnesses, and evaluated a novel model for predicting early-stage IGT and the “high-normal state.”

## Materials and Methods

### Materials

8-iso Prostaglandin F2α (8-iso-PGF_2α_), 8-iso-PGF_2α_-d_4_, (±)5-hydroxyeicosatetraenoic acid (5-HETE), (±)12-HETE, 15*(S)*-HETE, 13*(R*,*S)*-hydroxy-9Z, 11E-octadecadienoic acid (13-*(Z*,*E)*-HODE), 9*(R*,*S)*-*(Z*,*E)*-HODE, and 13*(R*,*S)*-*(Z*,*E)*-HODE-d_4_ were obtained from Cayman Chemical Company (MI, USA). 9*(R*,*S)*-*(E*,*E)*-HODE, 13*(R*,*S)*-*(E*,*E)*-HODE, 10*(R*,*S)*-*(Z*,*E)*-HODE, and 12*(R*,*S)*-*(Z*,*E)*-HODE were obtained from Larodan Fine Chemicals AB (Malmo, Sweden). Other materials were used at the highest grade that was commercially available.

### Subjects and sample processing

**Fig 1:**
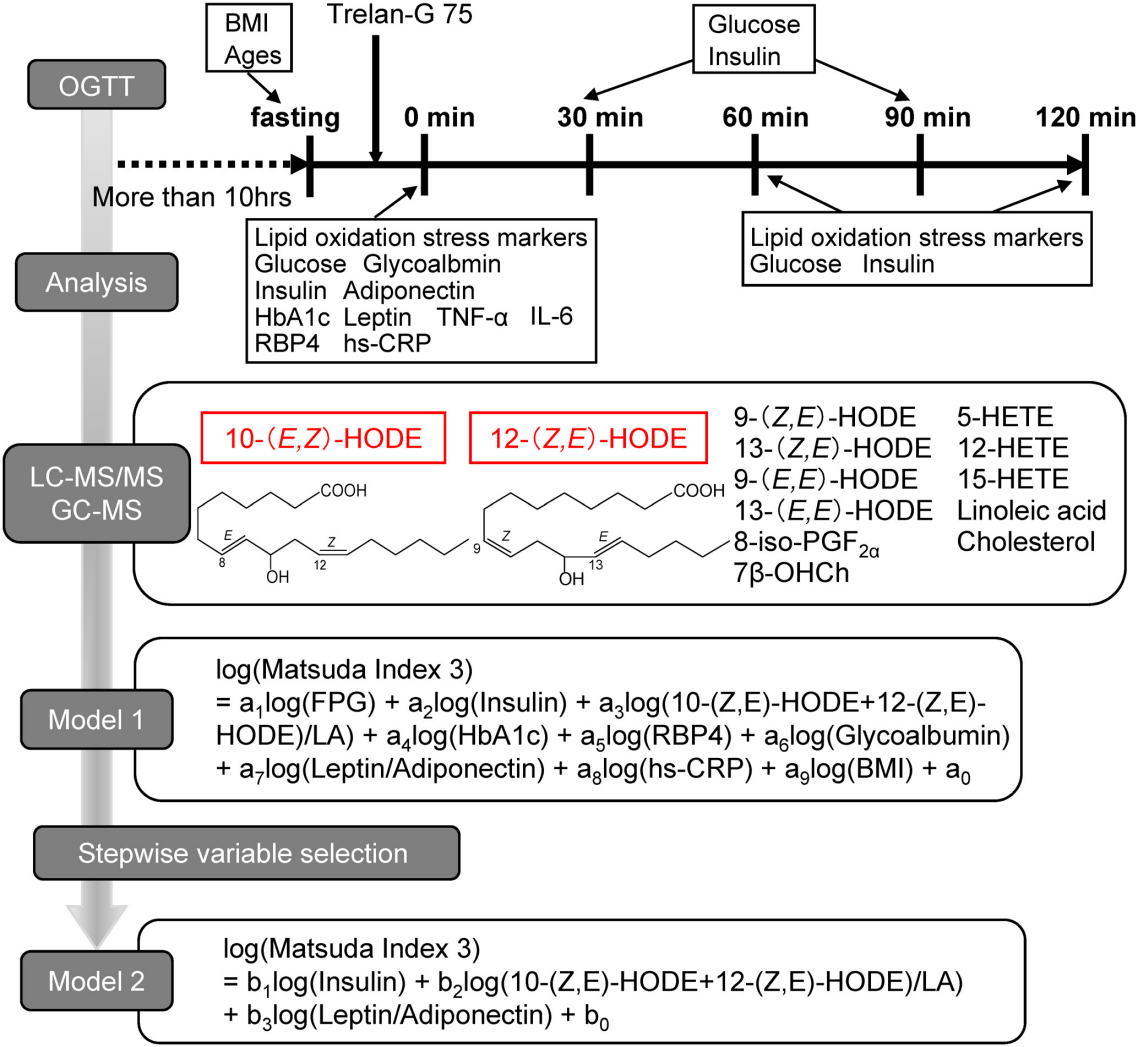
The study design, oral glucose tolerance test, and statistical analysis protocol.

We randomly enrolled 57 healthy volunteers, who had no history of any diseases. A 75 g-OGTT was performed for 120 min after >10 h of fasting, with blood collected every 30 min in tubes containing ethylenediaminetetraacetic acid disodium salt (EDTA–2Na) (Fig 1). As previously described [21], plasma and erythrocytes were separated immediately after collection via centrifugation at 1,500 × *g* for 10 min at 4°C. The plasma was subsequently frozen and stored at –80°C until analysis. This study was approved by the institutional review boards of the National Institute of Advanced Industrial Science and Technology and Tokushima University. All subjects gave written informed consent after the purpose of this study was completely explained.

### Analysis of oxidative stress markers

Plasma levels of oxidative stress markers and their parent molecules were measured as previously reported [21,22]. Isoprostanes and HODEs were analyzed by using liquid chromatography-tandem mass spectrometry (Thermo Finnigan TSQ Quantum Discovery Max, Thermo Fisher Scientific, CA, USA) after both reduction with triphenylphosphine and saponification with potassium hydroxide. The LA parent molecule was analyzed by using a gas chromatograph (GC 6890 N, Agilent Technologies, Palo Alto, CA, USA) that was equipped with a quadrupole mass spectrometer (5973 Network, Agilent Technologies).

### Detecting other biomarkers

HbA1c, glucose, insulin, leptin, adiponectin, RBP4, glycoalbumin, and high sensitivity-C-reactive protein (hs-CRP) were estimated using commercially available ELISA kits (HbA1c; RAPIDIA Auto HbA1c-L [Fujirebio Inc. (Tokyo, Japan)]; glucose, Cica liquid GLU J [KANTO CHEMICAL Co., Inc. (Tokyo, Japan)]; insulin, Lumipulse Presto Insulin [Fujirebio Inc. (Tokyo, Japan)]; leptin, Human leptin RIA kit [Millipore Co., Inc. (Tokyo, Japan)]; adiponectin, CircuLex Human adiponectin ELISA Kit CY-8050 [MBL Co. Ltd. (Nagano, Japan)]; RBP4, CircuLex Human RBP4 ELISA Kit [MBL Co. Ltd. (Nagano, Japan)]; glycoalbumin, Lucia GA-L [Asahi KASEI Pharma Co., Inc. (Tokyo, Japan)]; and hs-CRP, CircuLex Human HS-CRP ELISA Kit CY-8071 [MBL Co. Ltd. (Nagano, Japan)]).

### Statistical methods

Statistical analyses were performed on a Microsoft PC using SPSS software (version 14.0, SPSS Inc., Chicago, IL, USA). One-factor repeated measures design analysis of variance (ANOVA) was used to examine the effect of elapsed time from the glucose injection on each index. Significant effects were followed by Tukey's honestly significant difference multiple comparisons, and correlations were analyzed with the Pearson test. Data were expressed as mean ± standard deviation, and *p*-values of <0.05 were considered statistically significant.

We developed a multiple linear regression model to predict the Matsuda Index 3, as a function of 9 physiological variables (Model 1, Fig 1). The 9 variables were FPG, fasting levels of 10- and 12-*(Z*,*E)*-HODE/LA, HbA1c, RBP4, glycoalbumin, leptin/adiponectin, hs-CRP, and body mass index (BMI). These variables were used because they were significantly correlated with glucose levels at both 60 min and 120 min after the administration of glucose (*p* < 0.01) or with the Matsuda Index 3 (*p* < 0.05). Logarithmic transformation was applied to all variables to achieve normality before the analysis.

Moreover, we performed stepwise variable selection analysis for the 9 variables that we used as explanatory variables in Model 1. The criterion to add or delete a variable in the model was based on F-statistics, with a critical *p*-value of 0.05 (we refer to this as Model 2, Fig 1). Logarithmic transformation was applied to all variables to achieve normality before the analysis. We determined the cut off levels for FPG and the estimated Matsuda Index 3 value using the models to detect IGT and insulin resistance, and then calculated the sensitivity and specificity of the detection. The protocol for this study and the equations for the models are summarized in Fig 1.

## Results

### Characterization of subjects using the OGTT

**Fig 2:**
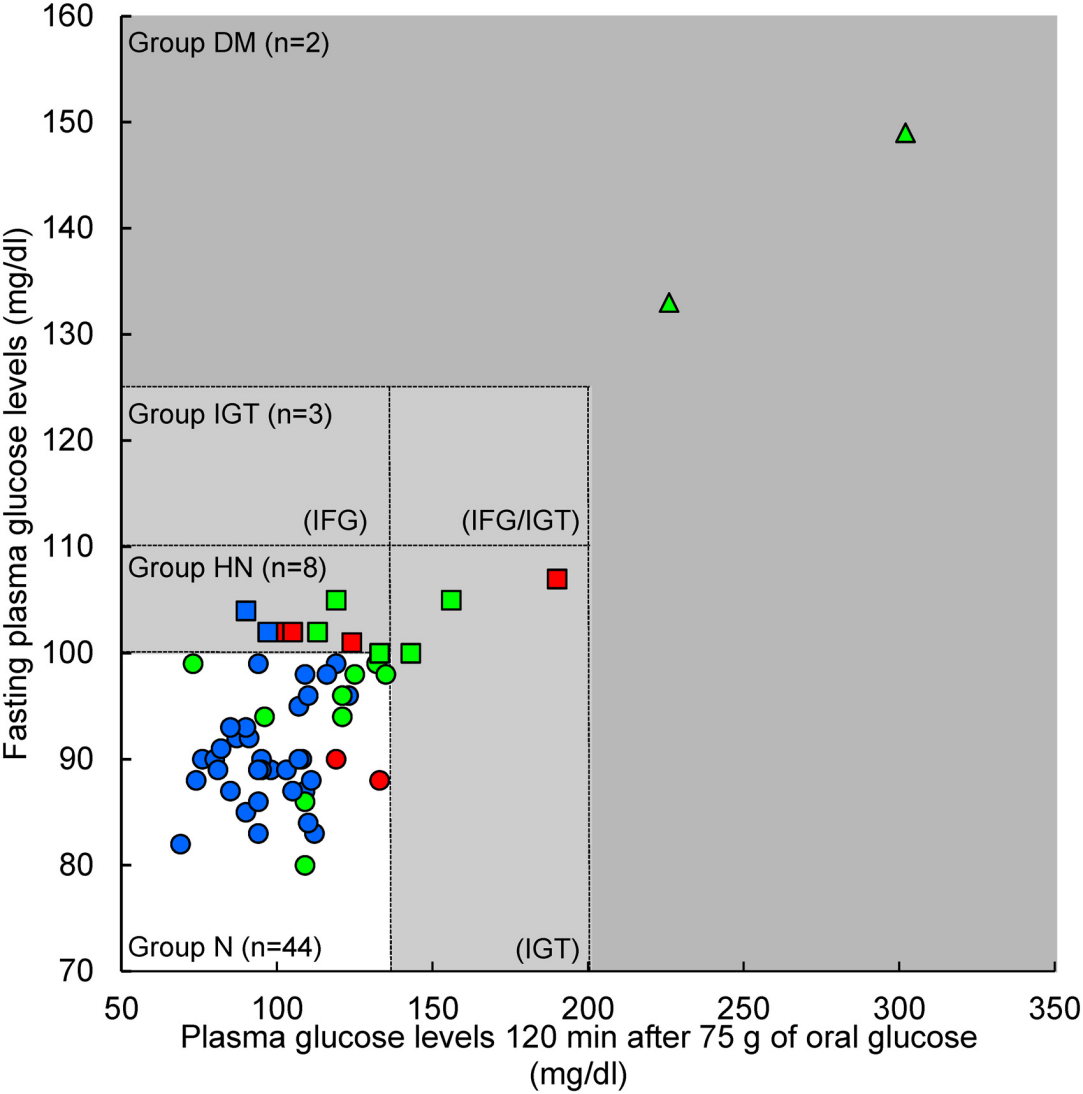
Classification of glucose tolerance using the oral glucose tolerance test. Circle, Group N (normal); square, Group HN+IGT (“high-normal” and impaired glucose tolerance); triangle, Group D (diabetic). Blue, normal insulin resistance; red, borderline insulin resistance; green, insulin resistance determined by homeostasis model assessment of insulin resistance and Matsuda Index 3.

The main characteristics and metabolic parameters of the subjects after the 75-g OGTT are shown in Table 1 and Fig 2. Among the 57 volunteers, 44 were characterized as normal (Group N), 11 as “high-normal” (fasting glucose levels of 100–109 mg/dL) with IGT (Group HN+IGT), and 2 as diabetic (Group D). It was surprising that 2 patients with diabetes were included among the volunteers, although the Japan Diabetes Society has reported that approximately 25–40% of “high-normal” subjects develop pre-diabetes and diabetes. Therefore, we considered “high-normal” and IGT subjects as a single group. There were significant differences between Group HN+IGT and Group N in their height and weight, although not in their BMI. The levels of HbA1c, RBP4, glycoalbumin, and hs-CRP tended to increase with reduced glucose tolerance, as indicated by the glucose levels. Fasting levels of RBP4 and glycoalbumin in Group HN+IGT were significantly higher than those from Group N. In contrast, fasting levels of adiponectin, leptin, and insulin in Group HN+IGT were not significantly different from those in Group N.

**Table 1:** Characteristics of subjects at entry into the oral glucose tolerance test.

		Group N	Group HN+IGT	Group D
		Normal	High-Normal^d^ Borderline Diabetic (IGT)	Diabetes
***N***		44	11	2
**Gender (M / F)**		17 / 27	10 / 1	2 / 0
**Age (years)**		37.8 ± 7.3	45.8 ± 9.2**	43.0 ± 8.5
**Height (cm)**		163.3 ± 8.3	170.5 ± 5.0**	173.0 ± 9.9
**Weight (kg)**		61.3 ± 12.3	71.5 ± 9.3*	83.0 ± 9.9**
**BMI (kg/m^2^)**		22.9 ± 4.1	24.6 ± 2.3	27.7 ± 0.1
**HbA1c (%)**		5.2 ± 0.3	5.3 ± 0.3	6.8 ± 0.5**
**RBP4 (μg/ml)**		15.1 ± 4.6	20.3 ± 2.9**	25.7 ± 5.7**
**Glycoalbumin (%)**		14.7 ± 2.1	17.2 ± 3.3**	20.4 ± 2.6**
**Adiponectin (μg/ml)**		7.8 ± 3.7	6.7 ± 2.8	5.2 ± 0.3
**Leptin (ng/ml)**		7.7 ± 4.6	6.0 ± 2.1	6.4 ± 0.7
**hs-CRP (μg/ml)**		0.7 ± 0.9	1.4 ± 1.8	3.1 ± 0.2**
**Glucose (mg/dl)**	0 ^a^	90.9 ± 5.1	102.7 ± 2.2**	141.0 ± 11.3**
	60 ^b^	124.7 ± 32.5	179.5 ± 43.1**	259.5 ± 2.1**
	120 ^c^	101.7 ± 16.9	124.7 ± 29.5**	264.0 ± 53.7**
**10- and 12-(Z,E)- HODE/LA (μmol/mol)**	0	0.7 ± 0.6	1.1 ± 0.9	1.9 ± 0.2**
	60	0.6 ± 0.4	0.7 ± 0.5	1.6 ± 0.4**
	120	0.6 ± 0.5	1.0 ± 1.4	2.1 ± 1.0**
**Insulin (μU/ml)**	0	6.0 ± 1.0	7.8 ± 5.0	9.6 ± 1.9
	60	61.7 ± 55.9	69.2 ± 38.0	49.0 ± 18.7
	120	48.3 ± 44.7	51.9 ± 29.9	57.9 ± 17.7
**L / A**		1.2 ± 1.0	1.0 ± 0.6	1.2 ± 0.1
**HOMA—IR**		1.4 ± 0.9	2.0 ± 1.3	3.4 ± 0.9**
**Matsuda index 3**		8.9 ± 5.3	5.8 ± 4.6	2.8 ± 0.0

Data are presented as mean ± standard deviation. One-facter completely randomized design analysis of variance (ANOVA) was used to examine the main effect of subject group on each index.

Shignificant effects were followed by Tukey's HSD multiple comparisons.

^a^, fasting

^B^, 60 min after 75g of oral glucose

^C^, 120 min after 75 g of oral glucose

^d^, fasting sugar, 100–109 mg/dl (defined by the Japan Diabetes Society)

Abbreviations: BMI, body mass index; RBP4, Retinol-Binding Protein 4; hs-CRP, high sensitivity C—reactive protein; HOMA-IR, homeostasis model assessment of insulin resistance; L/A, Leptin/Adiponectin

*p<0.05

**<0.01 compared with Group N.

**Fig 3:**
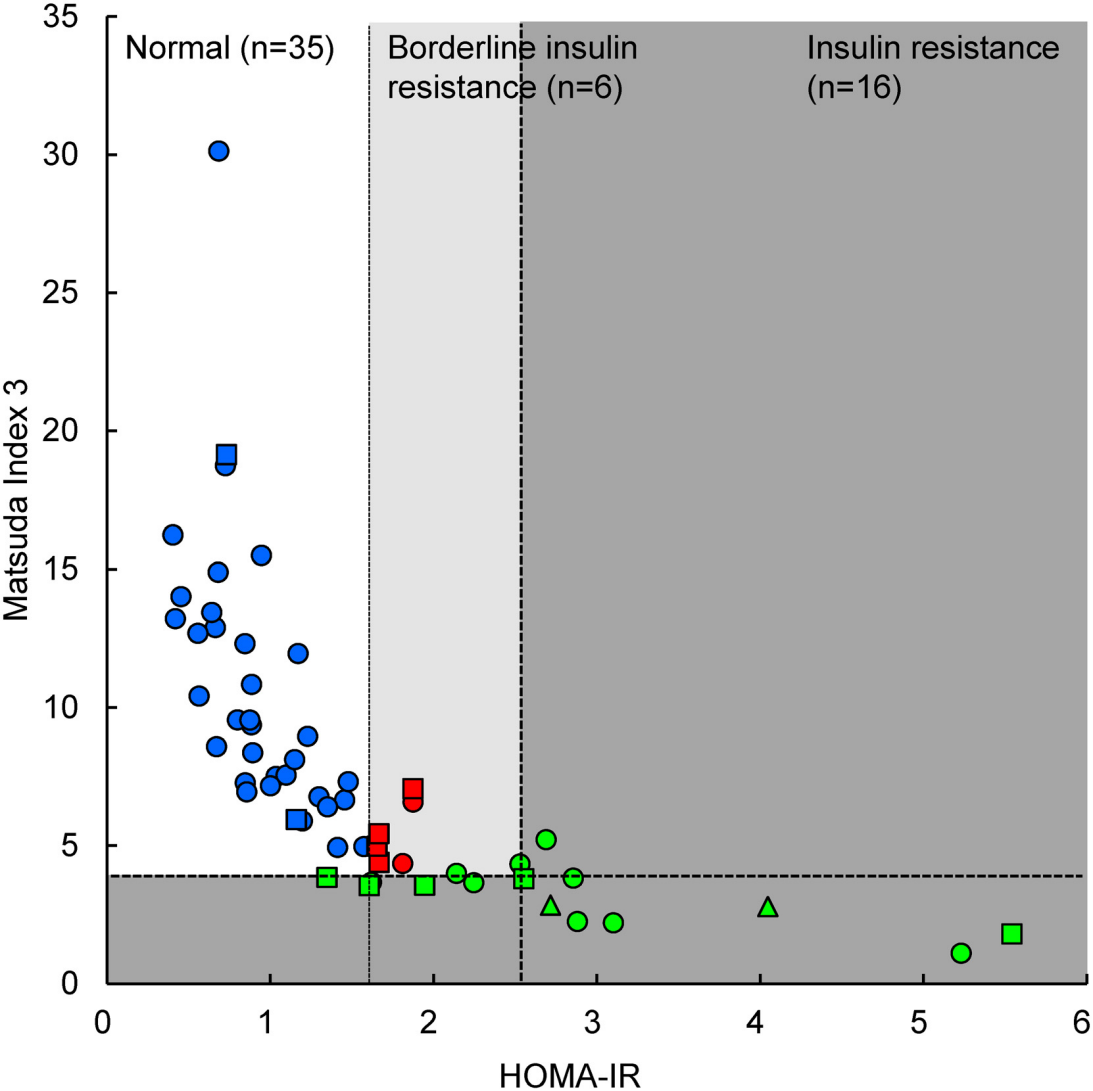
Classification of insulin resistance using the oral glucose tolerance test. Blue, normal insulin resistance; red, borderline insulin resistance; green, insulin resistance determined by homeostasis model assessment of insulin resistance and Matsuda Index 3. Circle, Group N (normal); square, Group HN+IGT (“high-normal” and impaired glucose tolerance); triangle, Group D (diabetic).

The HOMA-IR and Matsuda Index 3 have both been proposed as indices for insulin resistance and homeostasis assessment, although each relies on unique criteria. Previous studies [2,23] have reported that insulin resistance is defined by a HOMA-IR index >2.5, while insulin resistance is defined as a Matsuda Index <4. Fig 3 shows the classification of insulin resistance in this study, where 16 subjects were defined as insulin resistant and 6 were classified as borderline insulin resistant, using the above classifications. We found that 9 subjects in Group N and 5 subjects in Group HN+IGT were diagnosed as having abnormal insulin resistance (Figs 2 and 3). Moreover, 2 subjects in Group N and 4 subjects in Group HN+IGT had borderline insulin resistance.

### Correlation of 10- and 12-(*Z*,*E*)–HODE/LA and glycometabolism markers with glucose tolerance and insulin resistance index

**Fig 4:**
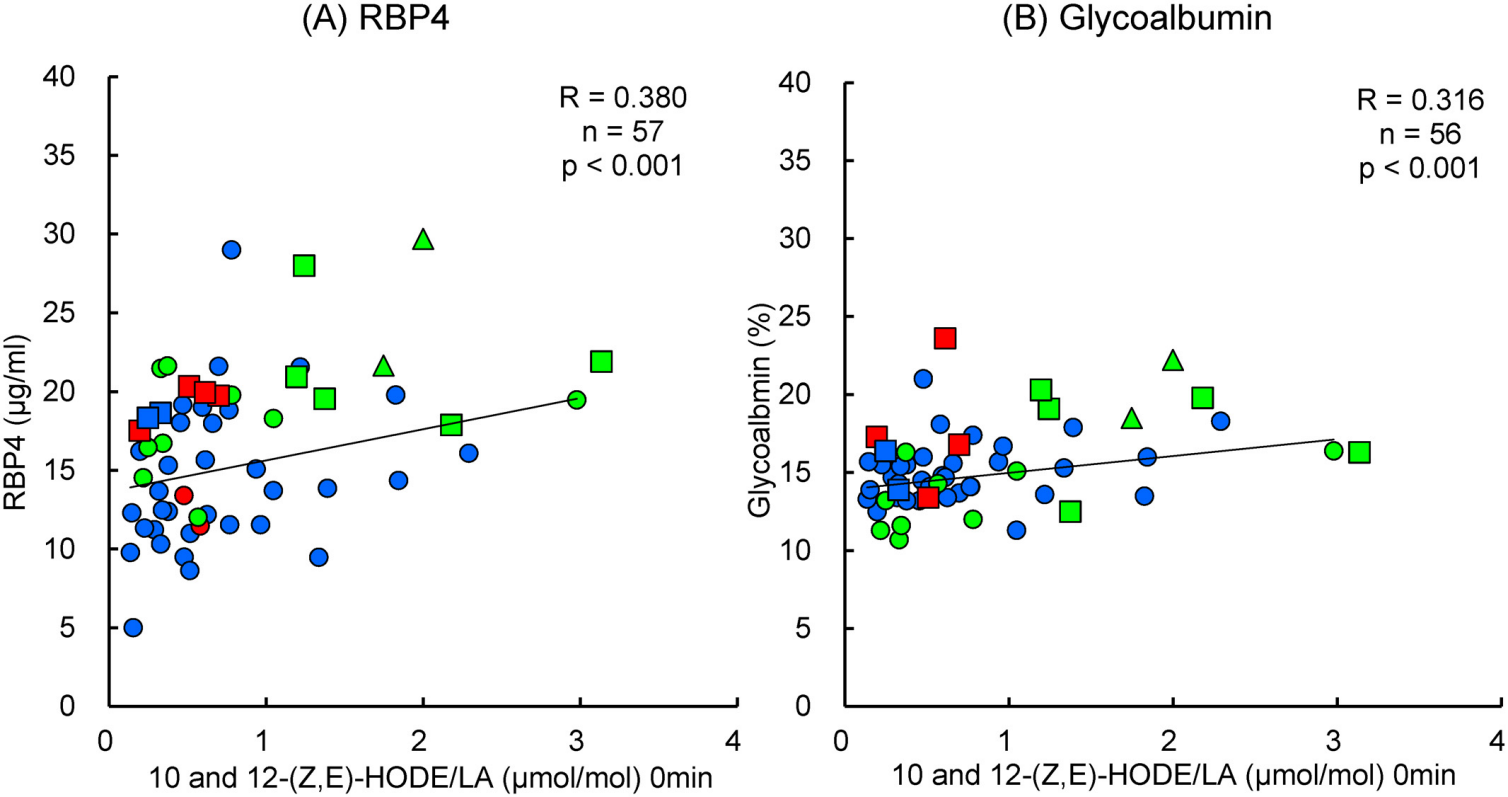
Correlation of 10- and 12-*(Z*,*E)*-HODE fasting plasma levels with (A) RBP4 and (B) glycoalbumin. Blue, normal insulin resistance; red, borderline insulin resistance; green, insulin resistance determined by homeostasis model assessment of insulin resistance and Matsuda Index 3. Circle, Group N (normal); square, Group HN+IGT (“high-normal” and impaired glucose tolerance); triangle, Group D (diabetic). 10- and 12-(*Z*,*E*)-HODE, 10- and 12-(*Z*,*E*)-hydroxyoctadecadienoic acid; RBP4, retinol binding protein 4.

We analyzed the correlations of glucose tolerance and insulin resistance with the fasting levels of 10- and 12-*(Z*,*E)*-HODE/LA, RBP4, glycoalbumin, adiponectin, leptin, and hs-CRP. Glucose tolerance was estimated using HbA1c, FPG, and glucose levels at 60 min and 120 min after the glucose administration. Insulin resistance was estimated using fasting plasma insulin, insulin levels during the OGTT, HOMA-IR, and Matsuda Index 3. 10- and 12-*(Z*,*E)*-HODE/LA, RBP4, hs-CRP, and adiponectin were significantly correlated with both glucose tolerance and insulin resistance (Table 2). 10- and 12-*(Z*,*E)*–HODE/LA, glycoalbumin, and hs-CRP were also well correlated with glucose tolerance and HbA1c. Interestingly, these markers were not correlated with insulin, nor with the insulin resistance indices (HOMA-IR and Matsuda Index 3). However, RBP4, adiponectin, and leptin/adiponectin were correlated with HOMA-IR and the Matsuda Index 3, and 10- and 12-*(Z*,*E)*–HODE/LA, RBP4, and, glycoalbumin were significantly correlated with glucose levels during the OGTT. Fig 4 shows the correlation between fasting plasma levels of 10- and 12-*(Z*,*E)*–HODE/LA and RBP4 (Fig 4A, *p* < 0.001), as well as glycoalbumin (Fig 4B, *p* < 0.001), which are both prominent biomarkers for the early detection of diabetes [5,6]. As 10- and 12-*(Z*,*E)*-HODE/LA alone can predict glucose tolerance, although not early-stage insulin resistance, we developed an algorithm to detect pre-diabetes using the above-mentioned markers.

**Table 2:** Correlations of the fasting plasma levels of 10- and 12-*(Z*,*E)*-HODE/LA, RBP4, glycoalbumin, hs-CRP, adiponectin, leptin, and L/A with glucose, insulin, HbA1c, Matsuda Index 3, and HOMA-IR during the OGTT.

		0min			60min		120min		Index	
		Glucose	Insulin	HbA1c	Glucose	Insulin	Glucose	Insulin	Matsuda Index 3	HOMA-IR
**0min**	**10- and 12-(ZE)-HODE/LA**	r = 0.478	r = 0.080	r = 0.320	r = 0.480	r = 0.107	r = 0.427	r = 0.086	r = - 0.268	r = 0.167
		p < 0.0001**	p = 0.553	p = 0.015*	p < 0.0001**	p = 0.428	p < 0.001**	p = 0.523	p = 0.044^#^	p = 0.214
		n = 57	n = 57	n = 57	n = 57	n = 57	n = 57	n = 57	n = 57	n = 57
	**RBP4**	r = 0.612	r = 0.420	r = 0.243	r = 0.492	r = 0.084	r = 0.492	r = 0.134	r = - 0.430	r = 0.445
		p < 0.0001**	p = 0.001**	p = 0.068	p < 0.0005**	p = 0.533	p < 0.0005**	p = 0.321	p < 0.001^##^	p < 0.001**
		n = 57	n = 57	n = 57	n = 57	n = 57	n = 57	n = 57	n = 57	n = 57
	**Glycoalbumin**	r = 0.541	r = 0.018	r = 0.468	r = 0.498	r = - 0.062	r = 0.480	r = - 0.134	r = - 0.072	r = 0.132
		p < 0.0001**	p = 0.895	p < 0.0001**	p < 0.0005**	p = 0.649	p < 0.0005**	p = 0.324	p = 0.597	p = 0.331
		n = 56	n = 56	n = 56	n = 56	n = 56	n = 56	n = 56	n = 56	n = 56
	**hs-CRP**	r = 0.426	r = 0.177	r = 0.403	r = 0.396	r = 0.143	r = 0.387	r = 0.208	r = - 0.311	r = 0.233
		p = 0.001**	p = 0.187	p = 0.002**	p = 0.002**	p = 0.288	p = 0.003**	p = 0.121	p = 0.019^#^	p = 0.081
		n = 57	n = 57	n = 57	n = 57	n = 57	n = 57	n = 57	n = 57	n = 57
	**Adiponectin (A)**	r = - 0.266	r = - 0.366	r = - 0.112	r = - 0.329	r = - 0.313	r = - 0.294	r = - 0.420	r = 0.407	r = - 0.374
		p = 0.045^#^	p = 0.005^##^	p = 0.405	p = 0.013^#^	p = 0.018^#^	p = 0.026^#^	p = 0.001^#^	p = 0.002**	p = 0.004^##^
		n = 57	n = 57	n = 57	n = 57	n = 57	n = 57	n = 57	n = 57	n = 57
	**Leptin (L)**	r = - 0.216	r = 0.391	r = 0.028	r = - 0.039	r = 0.305	r = 0.022	r = 0.403	r = 0.318	r = 0.306
		p = 0.113	p = 0.003**	p = 0.840	p = 0.780	p = 0.023*	p = 0.876	p = 0.002**	p = 0.018^#^	p = 0.023*
		n = 55	n = 55	n = 55	n = 55	n = 55	n = 55	n = 55	n = 55	n = 55
	**Leptin/Adiponectin (L/A)**	r = - 0.035	r = 0.564	r = 0.147	r = 0.173	r = 0.570	r = 0.149	r = 0.672	r = - 0.485	r = 0.495
		p = 0.800	p < 0.0001**	p = 0.288	p = 0.211	p < 0.0001**	p = 0.283	p < 0.0001**	p < 0.0001^##^	p < 0.0001**
		n = 54	n = 54	n = 54	n = 54	n = 54	n = 54	n = 54	n = 54	n = 54

**, ^##^ p<0.01

*,^#^ p<0.05

### Selecting fasting plasma markers to predict glucose tolerance and insulin resistance

**Fig 5:**
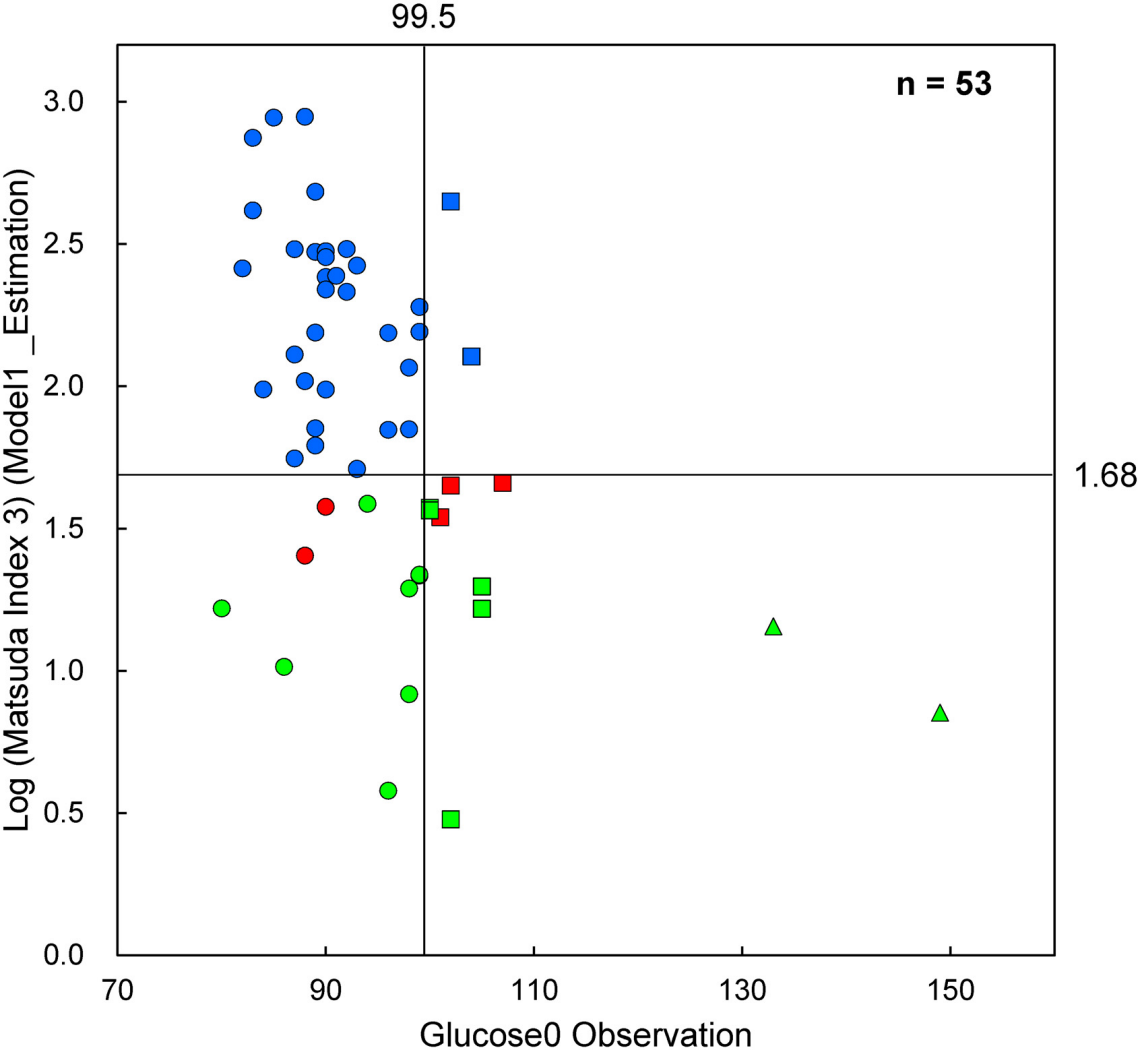
Predicting the risk of glucose tolerance and insulin resistance. Matsuda Index 3 is estimated using the 9 biomarkers that are shown in Model 1. Blue, normal insulin resistance; red, borderline insulin resistance; green, insulin resistance determined by homeostasis model assessment of insulin resistance and Matsuda Index 3. Circle, Group N (normal); square, Group HN+IGT (“high-normal” and impaired glucose tolerance); triangle, Group D (diabetic).

First, we designed an algorithm that could predict IGT and early stage insulin resistance using the fasting plasma markers, although this study did not include any subjects with isolated IFG and only 3 subjects with isolated IGT. As shown in Fig 2, the plasma glucose levels at 120 min after the OGTT were correlated with fasting glucose levels, and the Matsuda Index 3 was more sensitive to insulin resistance than HOMA-IR (Fig 3). The Matsuda Index 3 detected 12 subjects, while HOMA-IR detected 10 subjects, 8 of whom were detected using the Matsuda Index 3. This observation is understandable, because the Matsuda Index 3 is calculated using glucose and insulin levels during the OGTT, while HOMA-IR is calculated using fasting levels of both glucose and insulin. We then developed a multiple linear regression model to predict the Matsuda Index 3 using fasting plasma levels of the 9 biomarkers that correlated significantly with glucose levels at 60 min and 120 min after the OGTT, and/or the Matsuda Index 3 results. As shown in Fig 5, fasting levels of glucose (>99.5 mg/dL) and the Matsuda Index 3 estimation (<1.68) with the 9 biomarkers (Model 1), perfectly predicted glucose tolerance and insulin resistance, including borderline subjects.

### Detecting the risk of type 2 diabetes using insulin, leptin/adiponectin, and 10- and 12-(*Z*,*E*)-HODE/LA

**Fig 6:**
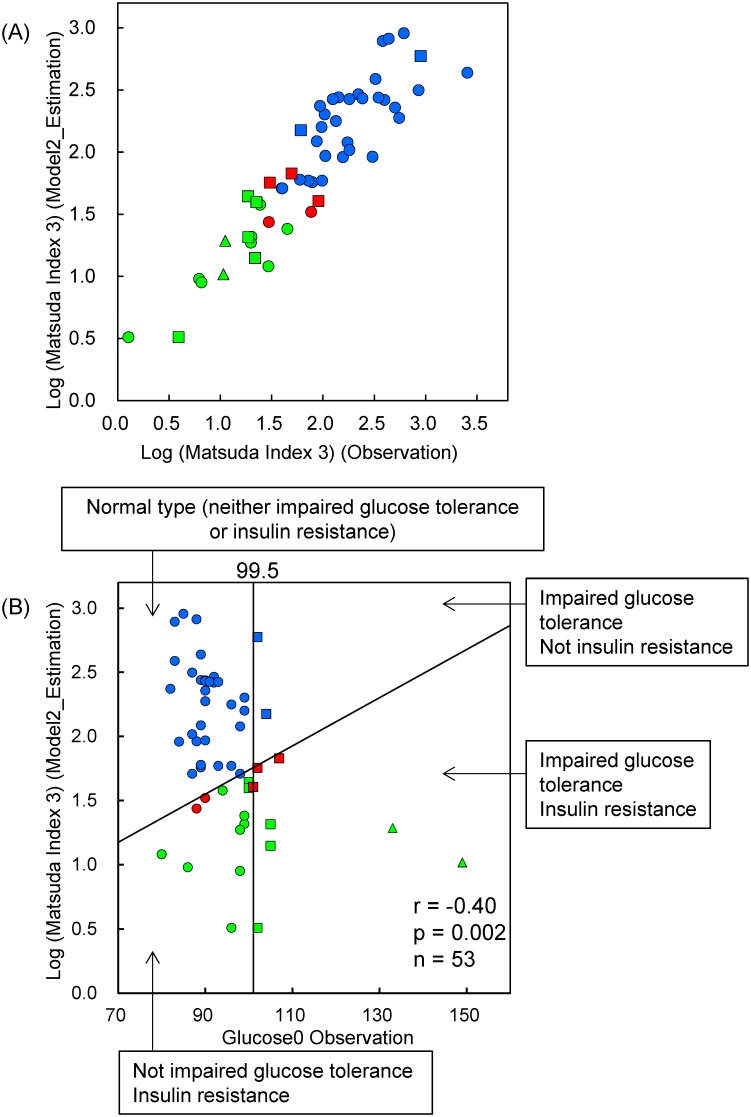
Selecting biomarkers for practical use. Matsuda Index 3 is estimated using 10- and 12-*(Z*,*E)*-hydroxyoctadecadienoic acid, insulin, and leptin/adiponectin (Model 2) (A), and the risk of glucose tolerance and insulin resistance is predicted using fasting levels of glucose and estimated Matsuda Index 3 (B). Blue, normal insulin resistance; red, borderline insulin resistance; green, insulin resistance determined by homeostasis model assessment of insulin resistance and Matsuda Index 3. Circle, Group N (normal); square, Group HN+IGT (“high-normal” and impaired glucose tolerance); triangle, Group D (diabetic).

To determine the practical use of the biomarkers, we performed stepwise variable selection analysis. Fasting levels of 10- and 12-*(Z*,*E)*-HODE/LA, insulin, and leptin/adiponectin were selected as explanatory variables for the multiple linear regression model (Model 2). As shown in Fig 6A, the selected markers accurately validated the Matsuda Index 3 estimates (r = 0.91, *p* < 10^−20^). Furthermore, FPG and the selected markers perfectly detected glucose tolerance and insulin resistance (sensitivity and specificity were 100%, Fig 6B).

## Discussion

Our multiple linear regression model (Model 2 in Fig 1), which consisted of 10- and 12-*(Z*,*E)*-HODE/LA, insulin, and leptin/adiponectin, perfectly predicted IGT and insulin resistance without using the OGTT. As it is generally accepted that IGT is largely due to insulin resistance, with IFG due to dysregulated gluconeogenesis, the OGTT is a useful tool for detecting the risk of both glucose tolerance and insulin resistance. However, as shown in Fig 2 and 9 subjects who had low levels of fasting and 120 min post-OGTT glucose also had abnormal levels of insulin resistance, despite the absence of isolated IGT or IFG. Therefore, the risk of insulin resistance cannot easily be determined using only the OGTT. Furthermore, a 75-g dose of glucose after fasting occasionally places a burden on the human body, especially for IFG and IGT subjects. However, we were also surprised to find that two diabetes subjects were included among our volunteers. Therefore, we analyzed the data without these patients, although our findings did not change. This fact is easily understood by considering Fig 6A, which shows the clear linearity between the estimated and observed values for the Matsuda Index 3. Although there are numerous methods for predicting type 2 diabetes, we believe this is the first method of risk evaluation that can evaluate both glucose tolerance and insulin resistance using only a few fasting biomarkers.

It is interesting that only 4 fasting plasma markers (10- and 12-*(Z*,*E)*-HODE/LA, insulin, and leptin/adiponectin) were able predict the risk of type 2 diabetes. However, 10- and 12-*(Z*,*E)*-HODE/LA had a good correlation with RBP4 and glycoalbumin levels, which is unsurprising, given that they have been frequently studied as biomarkers for glucose tolerance [5]. Interestingly, both markers correlated well with glucose levels, although not with insulin levels, during the OGTT, which indicates that they are appropriate for predicting glucose tolerance, although not for predicting insulin resistance. Clinical studies in children and adolescents have demonstrated that RBP4 has a role in obesity and the development of insulin resistance and type 2 diabetes [24]. Interestingly, Wu et al. reported that 8 weeks of fenofibrate treatment for insulin resistance in men with dyslipidemia reduced serum levels of RBP4 by 30%, which were correlated with reduced body weight and increased insulin sensitivity [25]. However, other studies have reported that this effect lacks clinical significance [26], which may be due to different genetic backgrounds, sex ratios, and age populations. HbA1c levels are also widely used as the gold standard for monitoring long-term glycemic control in patients with, given that HbA1c levels are associated with the development and progression of diabetic complications. However, as HbA1c reflects the conditions of HbA1c at 2–3 months before the analysis, it is not sensitive enough for the early detection of diabetes. In comparison, glycoalbumin provides a much earlier indication of failing glycemic control.

Leptin/adiponectin correlated well with insulin levels, although not with glucose levels, during the OGTT, which was expected. In this context, adiponectin and leptin are secreted exclusively by adipose tissue, and act as hormones with antagonistic effects. Adiponectin has anti-inflammatory and insulin-sensitizing properties, while leptin has pro-inflammatory effects. Thus, it is reasonable that L/A correlates very well with insulin levels during the OGTT.

We investigated the usefulness of our biomarker models using Akaike’s information criterion (AIC). In this context, lower AIC values indicate that a model has a better predictive ability. The AIC values for the models using 10 biomarkers (Fig 5) and 4 biomarkers (Fig 6B) were 30.33 and 20.07, respectively, which indicates that Model 2 (Fig 6B) has better predictive ability than Model 1 (Fig 5). Furthermore, we attempted to validate the importance of 10- and 12-(*Z*,*E*)-HODEs, and created three models that did not include these markers: Model 3 (insulin and L/A), Model 4 (insulin, L/A, and RBP4), and Model 5 (insulin, L/A, and glycoalbumin).The resulting AIC values were 32.6 (Model 3), 30.2 (Model 4), and 27.9 (Model 5). Therefore, it appears that 10- and 12-(*Z*,*E*)-HODEs are prominent biomarkers for the detection of early stage diabetes.

Hydroperoxyoctadecadienoic acids (HPODE) are formed by free-radical-mediated oxidation, and consist of 4 isomers (9- and 13-*(Z*,*E)*-HPODE and 9- and 13-*(E*,*E)*-HPODE), while singlet oxygen oxidizes LA through non-radical oxidation to form 4 unique isomers (9- and 13-*(Z*,*E)*-HPODE and 10- and 12-*(Z*,*E)*-HPODE). Thus, the 10- and 12-*(Z*,*E)*-HPODEs that we examined are specific oxidation products of singlet oxygen [21,22], and HPODEs are readily reduced to HODEs *in vivo*. Furthermore, it has been reported that singlet oxygen is produced *in vivo* by the reaction of hydrogen peroxide with hypochlorite, which is produced by myeloperoxidase that is secreted by activated phagocytes [27,28], by eosinophils through a peroxidase-catalyzed mechanism [29], and via bimolecular interactions between lipid peroxyl radicals [30]. Therefore, we speculate that neutrophils containing myeloperoxidase are recruited by hyperglycemia to adipose cells or β-cells, which results in the formation of singlet oxygen. This is considered the early stage of diabetes pathogenesis, and the normal response toward inflammation, before insulin secretion abnormality and insulin resistance are observed (adaptation). These hypotheses are supported by our recent studies [31, 32].

In an animal study [31], plasma levels of 10- and 12-*(Z*,*E)*-HODEs in a model of type 2 diabetes (Tsumura Suzuki Obese Diabetes mice) were significantly higher than those of control mice before IGT was observed. In addition, treating HaCaT cells with sub-lethal concentrations of 10- and 12-*(Z*,*E)*-HODEs, although not 9- and 13-*(Z*,*E)*-HODEs, caused resistance to hydrogen peroxide-induced oxidative damage, which indicates adaptive response [32]. Our previous study [21] also demonstrated that 10- and 12-*(Z*,*E)*-HODE, although not 9- and 13-HODE, were highly correlated with clinical values for diabetes and had diagnostic potential. Nevertheless, if these biomarkers are affected by the individual’s diet, it is likely that the levels of all HETEs/HODEs isomers would increase. In addition, results from animal models indicate that plasma levels of HODE are not affected by the daily diet, if it does not contain fortified coenzyme Q 9 or vitamin Es as supplements [15]. Furthermore, HODE levels did not fluctuate during the day, which suggests that they are not affected by diet (unpublished data from our laboratory). Thus, we believe that the 10- and 12-(*Z*,*E*)-HODEs markers in the present study were not influenced by the subjects’ diet. Therefore, HODE levels after 10 h of fasting may be appropriate for clinical use, as subjects’ biomarker levels are typically stable in the morning after fasting.

The present study clearly shows that multiple markers, including 10- and 12-*(Z*,*E)*-HODE/LA, insulin, and leptin/adiponectin, can be used for the early detection of diabetes. We have recently developed a second-generation prototype of a multi-marker analysis system, which is equipped with a CD-type microfluidic device [33] and an apparatus for measuring chemiluminescence. This device facilitates rapid assay of a single small-volume sample for numerous biomarkers. Although the present study indicates the diagnostic potential of multiple biomarkers within a limited number of subjects, these results must be validated, and we believe that this multi-marker analysis system will be useful for this validation. Furthermore, clinicians may be able to manage and/or advise subjects regarding their food and exercise habits (before the onset of diabetes) by evaluating their biomarker levels using this system. However, it is important to note that the diagnostic criteria vary throughout the world, and it would be interesting to determine whether these markers are accurate in other countries.
